# Identification of Oleanolic Acid as Allosteric Agonist of Integrin α_M_ by Combination of *In Silico* Modeling and *In Vitro* Analysis

**DOI:** 10.3389/fphar.2021.702529

**Published:** 2021-09-17

**Authors:** Lu Jin, Xiaoyu Han, Xinlei Zhang, Zhimin Zhao, Judith Ulrich, Tatiana Syrovets, Thomas Simmet

**Affiliations:** ^1^ Institute of Pharmacology of Natural Products and Clinical Pharmacology, Ulm University, Ulm, Germany; ^2^ School of Pharmaceutical Sciences, Sun Yat-Sen University, Guangzhou, China; ^3^ Department of Medicinal Chemistry, School of Pharmacy, Fourth Military Medical University, Xi’an, China

**Keywords:** oleanolic acid, integrin α_M_, allosteric agonist, metadynamics, free energy profile

## Abstract

Oleanolic acid is a widely distributed natural product, which possesses promising antitumor, antiviral, antihyperlipidemic, and anti-inflammatory activities. A heterodimeric complex formed by integrin α_M_ (CD11b) and integrin β_2_ (CD18) is highly expressed on monocytes and macrophages. In the current study, we demonstrate that the I domain of α_M_ (α_M_-I domain) might present a potential cellular target for oleanolic acid. *In vitro* data show that oleanolic acid induces clustering of α_M_ on macrophages and reduces their non-directional migration. In accordance with experimental data, molecular docking revealed that oleanolic acid binds to the α_M_-I domain in its extended-open form, the dominant conformation found in α_M_ clusters. Molecular dynamics simulation revealed that oleanolic acid can increase the flexibility of the α7 helix and promote its movement away from the N-terminus, indicating that oleanolic acid may facilitate the conversion of the α_M_-I domain from the extended-closed to the extended-open conformation. As demonstrated by metadynamics simulation, oleanolic acid can destabilize the local minimum of the α_M_-I domain in the open conformation partially through disturbance of the interactions between α1 and α7 helices. In summary, we demonstrate that oleanolic acid might function as an allosteric agonist inducing clustering of α_M_ on macrophages by shifting the balance from the closed to the extended-open conformation. The molecular target identified in this study might hold potential for a purposeful use of oleanolic acid to modulate chronic inflammatory responses.

## Introduction

Oleanolic acid is one of the most abundant terpenoids isolated from different edible plants as well as from many medicinal plants including *Panax ginseng* and *Eleutherococcus senticosus* (Liu, 1995; Jin et al., 2020). Oleanolic acid exhibited antitumor, antiviral, anti-inflammatory, antidiabetic, and hepatoprotective activities in various animal models (Liu, 1995; Ayeleso et al., 2017). Besides, oleanolic acid directly affects macrophages, their polarization and various functions. Thus, oleanolic acid inhibited the IL-10-induced macrophage polarization to the M2c subtype (Fujiwara et al., 2011) and induced macrophage-like differentiation of myeloid cell lines (Liu, 1995). The antitumor and immunoregulatory activities make oleanolic acid a promising lead for further development of drugs with increased bioavailability and potency. Some of oleanolic acid derivatives have been already evaluated in phase I/II clinical trials to treat advanced solid tumors and lymphoma (Ayeleso et al., 2017). Previously, it has been shown that oleanolic acid reduces reactive oxygen species levels (ROS), though, it does not scavenge ROS directly, but functions as an indirect ROS regulator by increasing the generation of antioxidant glutathione and the expression of key antioxidant enzymes (Ayeleso et al., 2017). The molecular mechanisms, especially the upstream targets of oleanolic acid are still largely obscure.

The integrin α_M_ (CD11b) is highly expressed on myeloid cells such as macrophages, neutrophils, and dendritic cells. It pairs with integrin β_2_ (CD18) to form the functional heterodimer α_M_β_2_ (CD11b/CD18, Mac-1) that controls processes necessary for immune responses, such as actin skeleton reorganization, cell adhesion, phagocytosis, and cell migration. Accordingly, integrin α_M_β_2_ is also important for pathogen removal and antigen presentation (Rosetti and Mayadas, 2016; Hu et al., 2020). In macrophages, specifically integrin α_M_ negatively regulates proliferation and alternative macrophage polarization in obesity (Zheng et al., 2015), reduces the number of tumor-infiltrating immunosuppressive myeloid cells, and stimulates proinflammatory antitumor macrophage polarization *in vivo* (Schmid et al., 2018; Panni et al., 2019).

Small molecule α_M_ agonists called leukadherins increase the α_M_β_2_-dependent myeloid cell adhesion, decrease their migration and accumulation in inflamed tissues, and reduce kidney damage in a mouse model of experimental nephritis (Maiguel et al., 2011). Hence, it was proposed that small molecule integrin agonists capable of transient decrease of immune cell accumulation in inflamed tissues might be beneficial for the treatment of chronic inflammatory diseases.

Similarities of functions of oleanolic acid and α_M_ agonists in macrophages led us to the hypothesis that integrin α_M_ may serve as a potential target of oleanolic acid. Indeed, in our previous work we presented evidence that integrin α_M_ might be a potential target of oleanolic acid (Jin et al., 2020). To further validate the hypothesis, the effect of oleanolic acid on the cell membrane clustering of α_M_ and macrophage motility were analyzed. In addition, molecular docking, molecular dynamics simulation, and metadynamics simulation were employed to analyze the potential molecular mechanisms of oleanolic acid-induced conformational changes of integrin α_M_.

## Materials and Methods

### Human Monocyte-Derived Macrophages

Monocytes were isolated from buffy coats from healthy volunteers by density gradient centrifugation and purified by adherence (Li et al., 2007). Monocytes were cultured in RPMI 1640 supplemented with 10% heat-inactivated fetal bovine serum containing 15 ng/ml recombinant human macrophage colony-stimulating factor (Miltenyi Biotec, Bergisch Gladbach, Germany) for 6 days to generate macrophages.

### Macrophage Treatment and Analysis

Integrin α_M_ clustering and migration were analyzed in macrophages grown on ibidi µ-bottom 8-well chamber slides (ibidi GmbH, Martinsried, Germany). Macrophages were stimulated with 1 μM oleanolic acid (Sigma) or 50 μg/ml fibrinogen for 60 min. Thereafter, cells were washed with cold PBS, fixed, permeabilized with 0.1% Triton X-100, and stained with FITC-conjugated CD11b antibody (Miltenyi Biotec). Images were acquired with a Nikon eclipse Ti microscope using a ×40 objective and NIS-elements microscope imaging software (Nikon Corporation, Tokyo, Japan). The fluorescence landscape was visualized with the Interactive 3D Surface Plot V2.4 plugin in ImageJ (National Institutes of Health, US), and clustering was identified and quantified as described (Faridi et al., 2013). Briefly, a 15% increase of baseline intensity was used as criterion for peak detection, and the number of peaks was used for quantification. Clusters in at least 15 cells/sample were calculated. To analyze non-directional macrophage migration in scratch assays, macrophages were treated with oleanolic acid (1 or 10 µM) or IL-4 (20 ng/ml, Miltenyi Biotec, Bergisch Gladbach, Germany) for 18 h. Images were acquired with a Nikon eclipse Ti microscope using a 10x objective and the cell-free areas at the beginning and the end of the experiment were calculated with the MRI Wound Healing tool in ImageJ.

Binding of oleanolic acid to human recombinant α_M_β_2_ (R&D Systems, Minneapolis, MN) was analyzed by differential scanning fluorimetry (Niesen et al., 2007) by using a LightCycler^®^ 480 (Hoffmann-La Roche, Basel, Switzerland). Briefly, 2 µM of α_M_β_2_ were mixed with 5x SYPRO Orange dye (Sigma), treated with either DMSO solvent or oleanolic acid, 10 µM, and subjected to thermal unfolding. Melting temperature (T_m_) was determined as the maximum of the first derivative of fluorescence emission as a function of temperature.

### Construction of the α_M_-I Domain/Oleanolic Acid Complex by Molecular Docking

Molecular docking was employed to investigate the interaction between the domain of α_M_ and oleanolic acid. The protein structure of the α_M_-I domain in extended-closed (PDBID: 1IDO) form meaning that the protein is partially open due to Mg^2+^ binding and extended-open (PDBID: 4M76) form meaning that the α_M_-I domain is fully open, activated by the presence of both, a divalent cation and the ligand C3d, were processed with Protein Preparation Wizard in Schrödinger suit and parameterized with OPLS-AA force field (Beard et al., 2013). A position restrained energy minimization was performed by default setting. The 3D conformations of ligands were generated by the LigPrep module in Schrödinger (2014) suite (Schrödinger 2014). Molecular docking was performed by using the InducedFit application in Schrödinger suite (Induced Fit Docking protocol, Glide, and Prime software packages) (Farid et al., 2006). Top scored conformation was used for further analysis.

### Unbiased Molecular Dynamics Simulation

Five different systems were prepared for unbiased molecular dynamics (MD) simulations (Supplementary Figure S1). In each system, the protein and ligands were parameterized with Amber 14SB force field (Maier et al., 2015) and generalized with Amber force field (Wang et al., 2004), respectively. The protein or complex were dissolved within TIP3P water box. The total atom number of all prepared systems was around 33,800, and the initial dimension of the system was around 74.6 Å × 68.7 Å × 71.5 Å.

To remove defects in structural models and to resolve the collision between atoms, a three-staged energy minimization protocol was employed. Firstly, all heavy atoms were position-restrained for 500 steps, and then the backbone atoms of protein and heavy atoms of ligands were position restrained for another 500 steps. Finally, the system was energy-minimized for another 1,000 steps without restriction. The energy-minimized system was gradually heated to 310 K in 100 ps with all heavy atoms restrained. Finally, the restraints were gradually removed in constant pressure environment (NPT) for a total of 2.5 ns. The production NPT run was performed at 310 K and 1 atm for at least 500 μs for every system.

The simulation time step was set to 2 fs, and the van der Waals interactions were calculated at every step. The long-range electrostatic interactions were calculated every 5 steps by the particle mesh Ewald (PME) method (Kolafa and Perram, 1992) with a grid size of about 1 Å and a tolerance of 10^−6^. The cut-off for the van der Waals interactions was set to 9 Å. The temperature and pressure were controlled using Langevin dynamics and the Langevin piston barostat method. The SHAKE method was used to restrain hydrogen atoms and the tolerance was set to 10^−8^. Atomic coordinates of all atoms were saved every 1 ps. To guarantee the reproducibility of key findings in MD simulations, five independent MD simulations were performed for each system using NAMD 2 (Phillips et al., 2020).

### Metadynamics Simulation

The conformational shift of the α7 helix was sampled with Metadynamics simulation (MetaD) using the equilibrated structure from production NPT run as the starting structure. To trace conformational change, the center of mass (COM) distance between Cα atoms of the N-terminus (residues 132–142) and α7 helix (residues 301–310) was defined as collective variable (CV). The grid’s boundary was set to 14.0 Å (lower boundary) and 30.0 Å (upper boundary), and the grid width was set to 0.2 Å. The hill weight and hill width was set to 0.01 and 1.0, respectively. To guarantee the reproducibility of key findings in MetaD simulation, three independent MD simulations were performed for each system using NAMD 2 (Phillips et al., 2020).

### Trajectory Analysis

Gromacs (Hess et al., 2008) and CARMA (Koukos and Glykos, 2013) were employed for trajectory analyses. After converting the amber topology to Gromacs with Acpype (Sousa da Silva and Vranken, 2012), the root-mean-square deviations (RMSD) and root-mean-square-fluctuations (RMSF) corresponding to the reference structure were calculated with Gromacs. The secondary structure of the protein was calculated by DSSP (Touw et al., 2015) and visualized with do_dssp in Gromacs.

The analysis of the dynamic behavior of key regions identified by RMSF analysis (regions with higher RMSF) in different systems was performed by PyEMMA (Scherer et al., 2015) as previous described (Jin et al., 2019). Briefly, the coordinations of Cα atoms in each region were used to define the initial model, and dimension of input coordinates was reduced by TICA (Time-Lagged Independent Component Analysis) on two dimensions. To converge the maximum likelihood estimation, the time step was set to 5.0 ns (1,000 steps) (Supplementary Figure S2A). Furthermore, the output of TICA was clustered into microstates using k-means clustering. The microstates were grouped to 5 metastable states by Perron-Cluster Cluster Analysis (PCCA++). The number of metastable states was selected according to spectral analysis and the Chapman-Kolmogorov test (Supplementary Figure S2B). The occupation of metastable states in each model was calculated by normalizing the frame numbers assigned to each metastable state with the total frame numbers.

### Residue Interaction Network Analysis

Residue Interaction Network analysis was conducted according to references (Hu et al., 2014; Li et al., 2019) by taking the non-adjacent amino-acid residues connected by non-covalent interactions, such as hydrogen bonds, salt bridge, or hydrophobic interactions, into consideration. The interactions between residues in each snapshot of MD trajectory were analyzed with RING (Martin et al., 2011), and the interactions with frequency higher than 0.05 were extracted to construct the initial residue interaction network. After filtering with degree and edge betweenness, the final network was constructed and used for further analysis.

### Statistical Analysis and Visualization

Data were analyzed using one-way ANOVA followed by Tukey post-hoc test with SigmaPlot 11.0 software (Systat Software Inc., San Jose, CA). Differences were considered significant at *p* < 0.05. The 3D coordinates of snapshots were visualized with PyMOL (Schrödinger 2015), and the line plots, as well as the histograms were plotted with Seaborn package (Waskom, 2017) in Python. The residue interaction network was analyzed and visualized by Cytoscape 3.8 (Shannon et al., 2003).

## Results

### Oleanolic Acid Induces Clustering of α_M_ on Macrophages and Reduces Their Non-directional Migration

**FIGURE 1:**
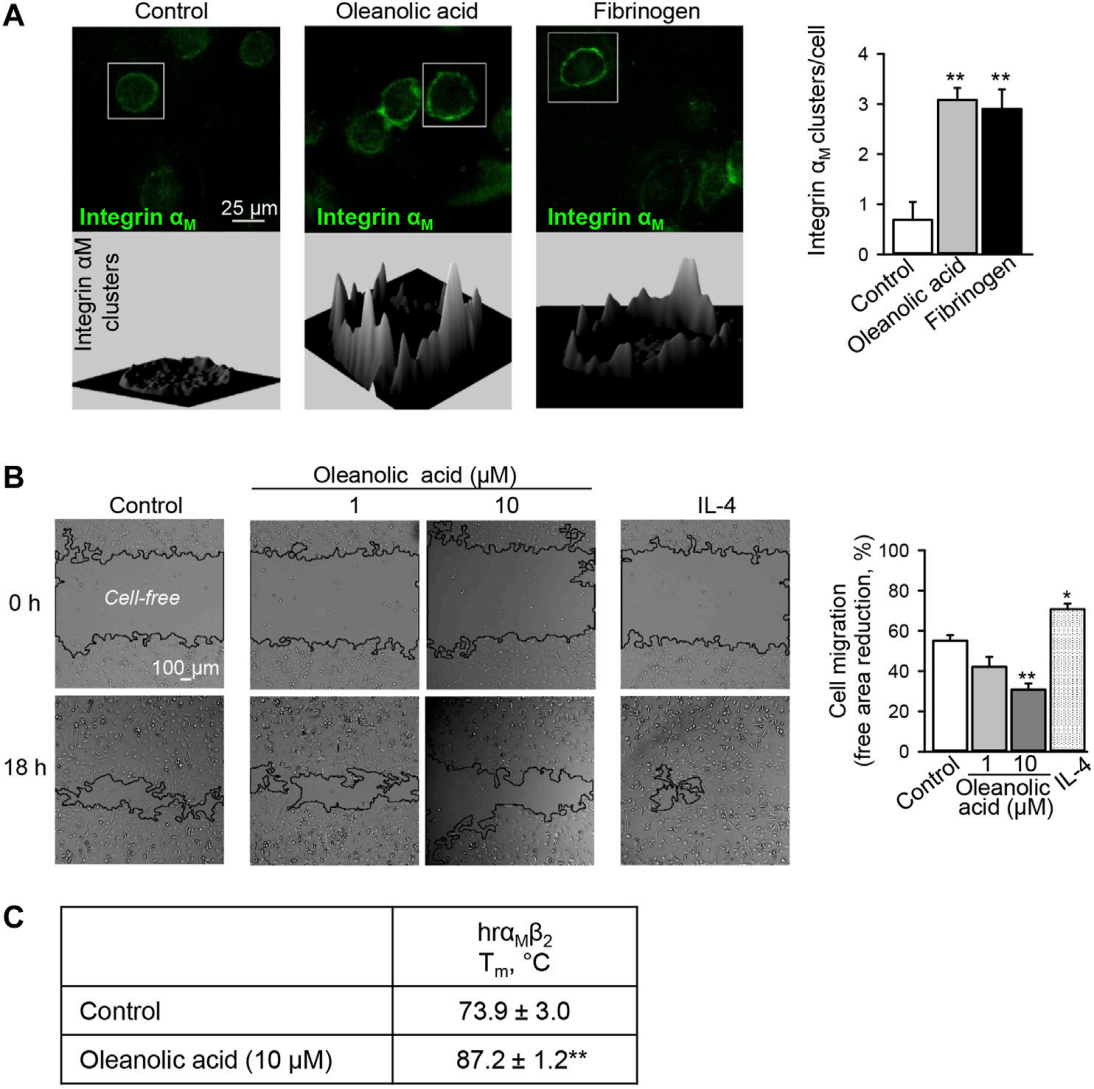
Oleanolic acid induces integrin α_M_ clustering on human macrophages and reduces their non-directional migration. **(A)** Oleanolic acid induces α_M_ clustering on M0 macrophages. Human monocyte-derived macrophages were treated with oleanolic acid (1 µM) or fibrinogen (50 μg/ml) for 1 h, and α_M_ was detected by fluorescence-labeled antibody followed by microscopy. The 3D landscape of α_M_ distribution was visualized with Interactive 3D Surface Plot plugin in ImageJ. The clusters per cell were calculated by analyzing at least 40 cells/sample. The results were obtained from at least 3 macrophage preparations. **(B)** Oleanolic acid decreases non-directed migration of macrophages. Migration of macrophages was evaluated in the scratch assay. Macrophages were treated with oleanolic acid (1 or 10 µM) or IL-4 (20 ng/ml) for 18 h, and the cell-free areas at *t* = 0 h and *t* = 18 h were calculated with the MRI Wound Healing tool in ImageJ. Data are from 4 macrophage preparations. **(C)** Binding of oleanolic acid to α_M_β_2_ as analyzed by differential scanning fluorimetry. Human recombinant α_M_β_2_ (2 µM) was treated with either DMSO solvent or oleanolic acid (10 µM) and subjected to thermal unfolding. Melting temperature (T_m_) was determined as the maximum of the first derivative of fluorescence emission as a function of temperature (*n* = 3). All data are mean ± SEM, *p < 0.05, **p < 0.01. One-way ANOVA followed by Tukey post-hoc test was employed for multi-group analysis. Representative images are shown.

As a result of activation, integrins cluster on cell membranes, recruit additional intracellular adaptor proteins causing enhanced cell adhesion (Michael and Parsons, 2020). In our previous study, we have observed that *Eleutherococcus senticosus* bark extract enriched in oleanolic acid decreases the migratory activity of macrophages (Jin et al., 2020), which may point to increased cell adhesion as has previously been shown for the α_M_β_2_ agonists, leukadherins (Maiguel et al., 2011). Also treatment of THP-1 monocytic cells with leukadherins induced α_M_β_2_ clustering (Maiguel et al., 2011). Therefore, we have microscopically analyzed the distribution of integrin α_M_ at the macrophage cell membrane. The α_M_ ligand fibrinogen was used as positive control. As expected, fibrinogen induced the clustering of α_M_ on macrophages. Similarly, clustering of α_M_ was observed after oleanolic acid treatment, which shares similar efficacy with fibrinogen **(** Figure 1A **)**.

Increased α_M_β_2_ clustering has been associated with increased adhesion and reduced cell motility (Maiguel et al., 2011). Hence, non-directional migration of macrophages was analyzed in the presence of oleanolic acid or IL-4. Similar to leukadherins, oleanolic acid decreased non-directional migration of macrophages (Figure 1B). Early studies have shown that different to leukadherins and oleanolic acid, fibrinogen does not affect non-directional migration of cells expressing α_M_β_2,_ though it is a chemoattractant for 293 cells overexpressing functional α_M_β_2_ heterodimer (Forsyth et al., 2001). This might indicate that conformational changes of α_M_ upon fibrinogen binding differ from those induced by leukadherins and oleanolic acid. In addition, in the presence of oleanolic acid full recombinant α_M_β_2_ showed a significant increase in T_m_ from 73.9 ± 3.0°C to 87.2 ± 1.2°C (*p* < 0.01) confirming the binding capacity of oleanolic acid to α_M_ (Figure 1C).

### Oleanolic Acid Selectively Binds to the Allosteric Pocket of the α_M_-I Domain in Its Extended-Open Conformation

With respect to ligand binding, integrins may exist in different confirmations, exposing either low- or high-affinity. In the low-affinity state, integrins adopt the so-called closed confirmation formed by the ligand-binding I and β-propeller domains of the α_M_ and the β-I-domain of the β_2_ subunit. In the high-affinity state, the ligand binding domain is open and accessible for a ligand (Rosetti and Mayadas, 2016). Therefore, we have analyzed binding of oleanolic acid to integrin α_M_, particularly to the ligand-binding α_M_-I domain by molecular docking. The allosteric pocket, was chosen as binding site for oleanolic acid based on its chemical structure. Two crystal structures describing the extended conformation of the α_M_-I domain, the extended-closed conformation (PDBID: 1IDO) and extended-open conformation (PDBID: 4M76), were extracted from the protein data bank and used to construct the initial model.

**FIGURE 2:**
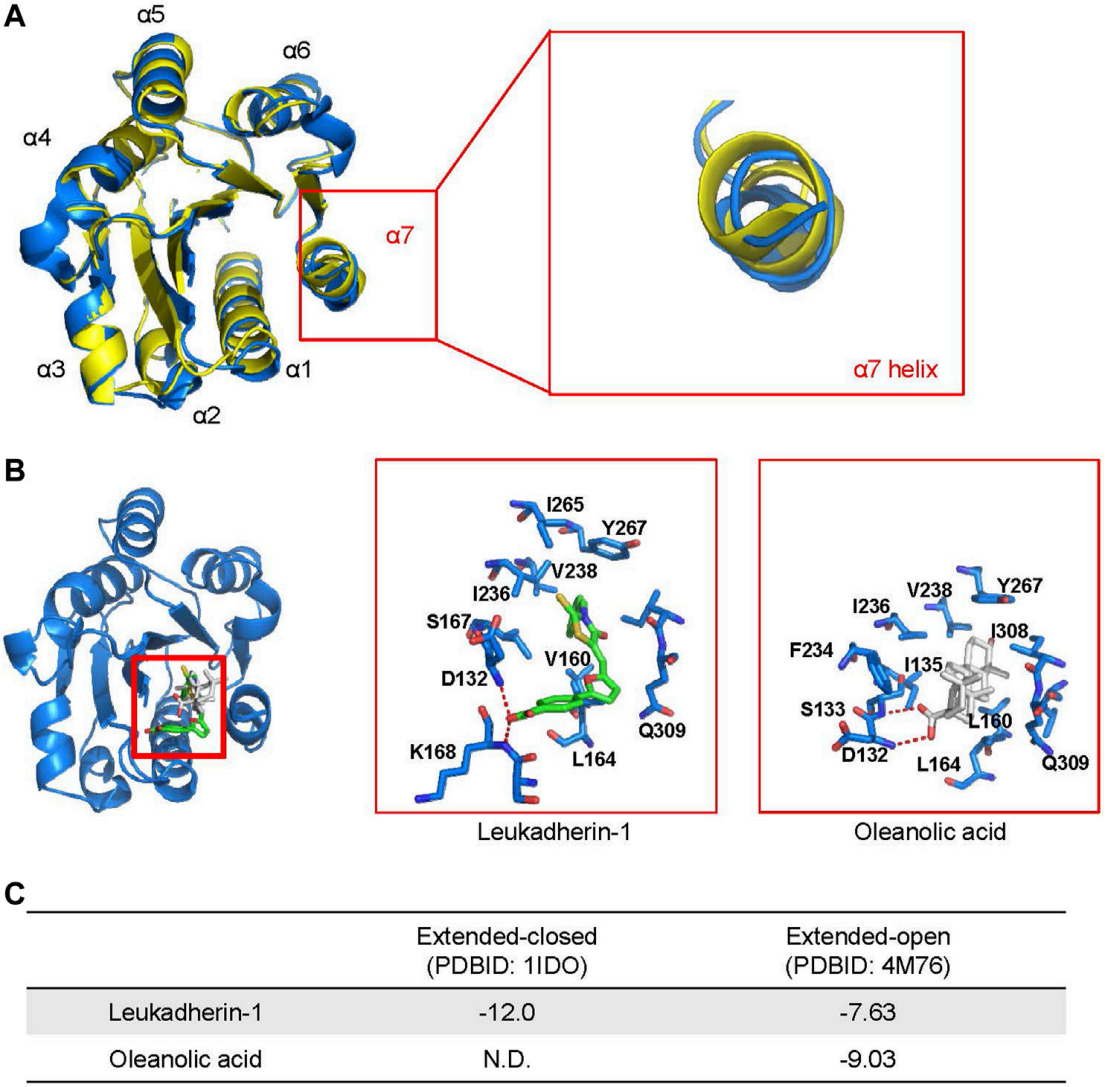
Oleanolic acid is predicted to fall into the allosteric site within the I domain of integrin α_M_ (α_M_-I domain) specifically in its extended-open conformation. **(A)** An overlapping of structures of the α_M_-I domain in the extended-closed (PDBID: 1IDO, colored yellow) and extended-open (PDBID: 4M76, colored blue) conformations. **(B)** The top-scored binding pose of leukadherin-1 and oleanolic acid binding to allosteric site of α_M_-I domain in its extended-open conformation. The carbon atoms of leukadherin-1 and oleanolic acid are colored green and white, respectively. **(C)** The predicted binding free energy of leukadherin-1 and oleanolic acid to the α_M_-I domain.

As shown in Figure 2A, the α7 helix of the α_M_-I domain adopts different conformation in its extended-closed and extended-open conformations. In the extended-open conformation, the α7 helix is slightly moved towards its C-terminus, which results in formation of a slightly enlarged allosteric binding site in the α_M_-I domain. Accordingly, the allosteric site shows now increased preference for a ligand with increased size or rigid scaffold. To validate the reliability of the established molecular docking model, leukadherin-1, an allosteric agonist of integrin α_M_, was used as a positive control. As shown in Figure 2B, leukadherin-1 falls into a narrow pocket close to the α7 helix, and stabilizes mainly through hydrophobic interactions with surrounding I265, V238, I236, Y367, and L164. The linear shape and the flexibility enable leukadherin-1 entering the inner pocket, which results in higher affinity to the α_M_-I domain in the extended-closed form (−12.0 Kcal/mol) compared to the extended-open form (−7.63 Kcal/mol) (Figures 2B,C). This finding is in accordance with literature data demonstrating that leukadherin-1 was able to stabilize the extended-closed form (Maiguel et al., 2011; Faridi et al., 2013). Differently, oleanolic acid cannot fit into the allosteric pocket in the extended-closed form, but shows promising predicted affinity to the allosteric pocket in the extended-open form (−9.03 Kcal/mol) (Figures 2B,C).

Interestingly, the results from molecular dynamics simulation showed that the effects of oleanolic acid and leukadherin-1 on the α_M_-I domain differ remarkably. As demonstrated by *in vitro* studies, leukadherin-1 functions as an allosteric agonist of the α_M_-I domain by stabilizing it in extended-closed form (Faridi et al., 2013). In accordance with published data, the results of our simulation showed that leukadherin-1 did affect neither the overall stability nor the α_M_-I domain nor the orientation of the α7 helix.

Molecular docking also demonstrates that compounds with a scaffold similar to oleanolic acid, such as ursolic acid and amyrin, exhibit in analogy to oleanolic acid preferences for the extended-open α_M_-I domain conformation (Supplementary Figures S3A,B). However, cholesterol that exhibits a smaller planar skeletal structure, binds with higher binding affinity to the α_M_-I domain in its extended-closed form. In addition, cholesterol, different to oleanolic acid, did not affect the stability of the α7 helix as analyzed by molecular dynamics simulation (Supplementary Figure S3) nor the direction of the α7 helix movement as analyzed by dynamics cross-correlation matrices (DCCMs) (Supplementary Figure S4).

### Oleanolic Acid Directs the Motion of the α7 Helix Away From the N-Terminus and Towards Its C-Terminus

To further investigate the stability of ligand binding in these complexes, a series of molecular dynamics simulations using the α_M_-I domain in extended-closed or extended-open conformations, with leukadherin-1 and oleanolic acid were performed. In agreement with the results from molecular docking, the binding free energy calculated from MM-PBSA (Supplementary Figure S5A **)** as well as the interaction analysis (Supplementary Figure S5B **)** showed that leukadherin-1 and oleanolic acid bind to the allosteric pocket of the α_M_-I domain with promising binding affinity. The binding poses of two compounds are stabilized mainly through hydrophobic interactions.

**FIGURE 3:**
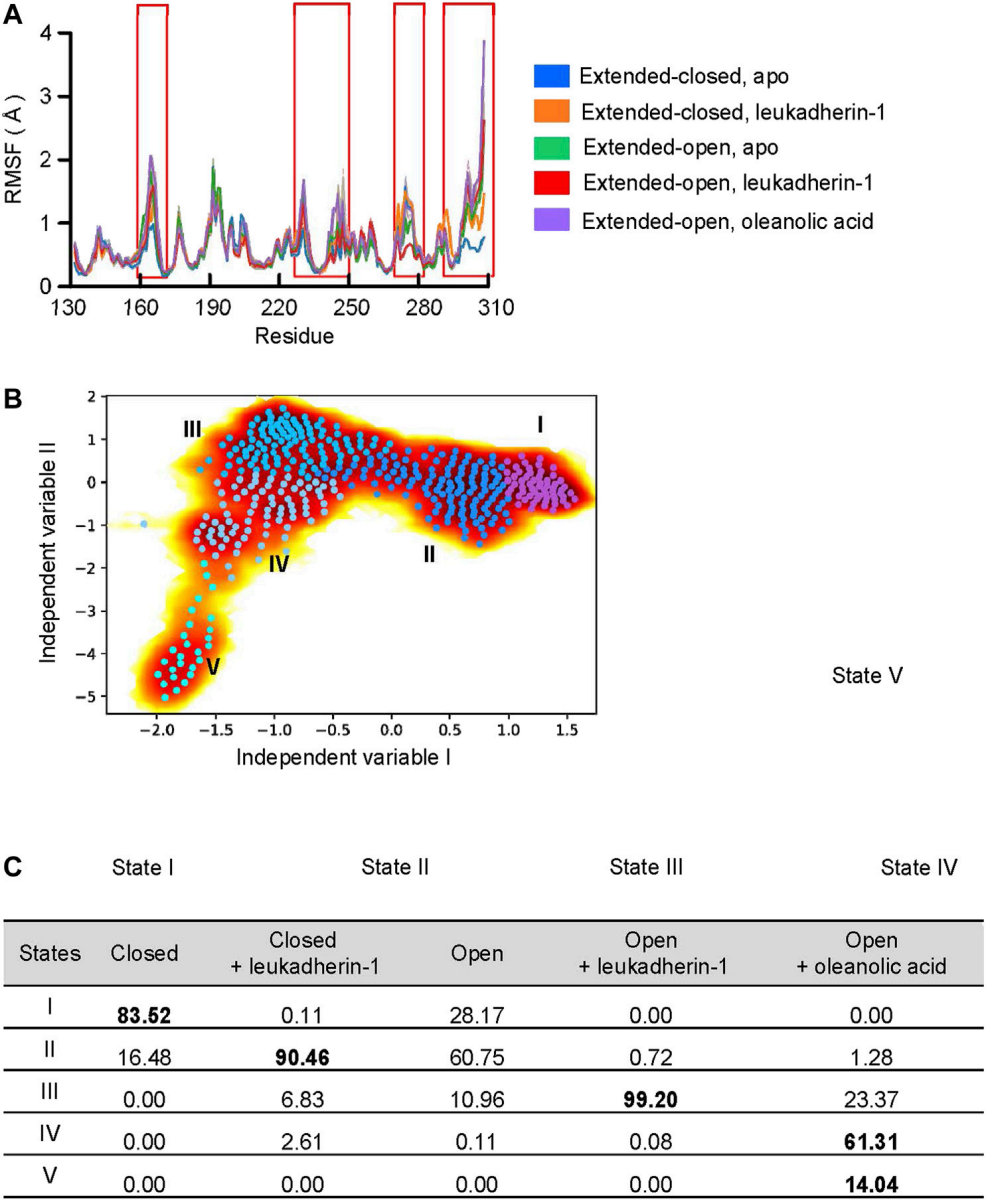
Oleanolic acid is able to induce a conformational shift of the α-7 helix away from the N-terminus. **(A)** The RMSF profile of five different models. The mean RMSF are plotted with thick lines, and the min/max are plotted as shadings. **(B)** Free energy profile from time-lagged independent component analysis (TICA) plot with microstates (dots). The microstates were clustered to five metastable states (I, medium orchid; II, dodger blue; III, deep sky blue; IV, light sky blue, and V, cyan). The representative structures of each metastable state are plotted in the lower panels; the residues in the α7 helix are highlighted with blue, orange, red, and magenta, respectively. **(C)** The distribution of metastable states in five different models. The highly populated populations are highlighted.

To validate, whether the ligands are able to affect the dynamic behavior, especially the long-time statistical conformational dynamics of the α_M_-I-domain, Markov state models (MSMs) were employed. MSMs identified five metastable states, which are characterized mainly by different orientation and conformation of the α7 helix (Figures 3A,B). Although the conformation of the α7 helix was slightly different in state I and state II, the center of mass (COM) distance between the N-terminus and the α7 helix was 15.9 Å in both metastable states (COM distance in 1IDO, 15.9 Å), indicating that both of them belong to the extended-closed conformation. In state III, the COM distance between the N-terminus and the α7 helix increased to 17.0 Å, which is similar to the crystal structure of the open-extended conformation of the α_M_-I domain (COM distance in 4M76, 16.8 Å). In state IV and state V, the COM distance between the N-terminus and the α7 helix are 20.6 Å and 19.9 Å, respectively.

According to the probability distribution, the α7 helix in extended-open conformation is gradually shifted to the extended-closed form in the absence of a ligand, which is characterized by the decreased population in state III, but increased population of states I and II. Leukadherin-1 showed little effect on the probability distribution or the conformational shift of α7. Different from other models, two unique states were detected in the α_M_-I domain complexed with oleanolic acid. The increased distance and the slightly shifted α7 helix indicate that oleanolic acid may switch the moving direction of the α7 helix away from its N-terminus (Figure 3C).

### Oleanolic Acid Increases the Flexibility and Decreases the Secondary Structure Stability of the α7 Helix in the α_M_-I Domain

**FIGURE 4:**
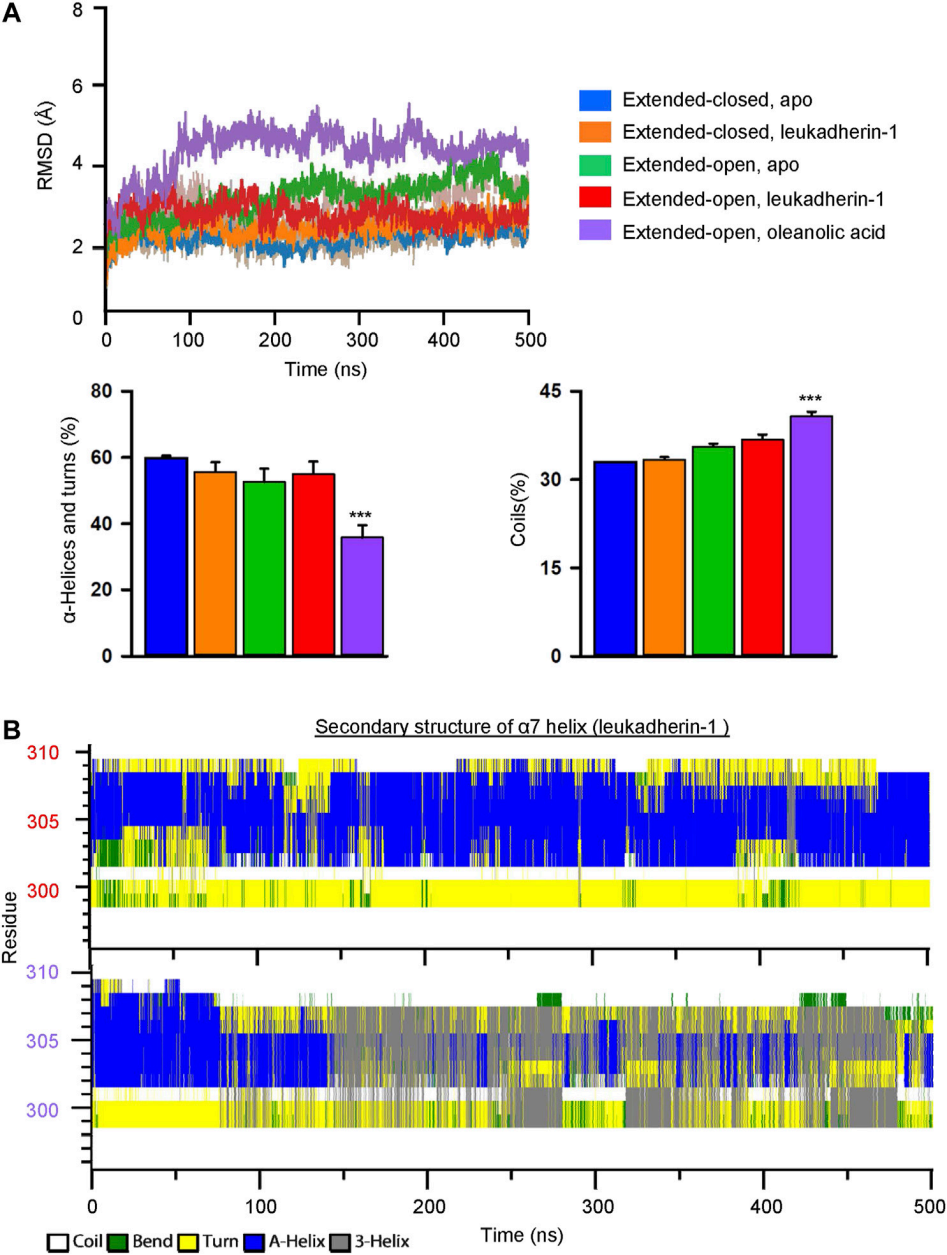
Oleanolic acid decreases the stability of secondary structures and increases the flexibility of the α7 helix in the α_M_-I domain. **(A)** The RMSD of the α7 helix in five different models. The mean values of RMSD in different models are plotted in lines, and the min/max are plotted in shadings. Graphs show that oleanolic acid decreases the percentage of α-helices and turns, but increases the percentage of coils in the α7 helix along with the simulation. The secondary structure profile was calculated by do_dssp application in Gromacs, and the data are presented as mean ± SEM from 5 independent simulations, ***p < 0.001. **(B)** The represented secondary structure profile of the α_M_-I domain in complex with leukadherin-1 (upper) and oleanolic acid (lower) along with conventional molecular dynamics simulation.

To investigate the effect of leukadherin-1 and oleanolic acid on the flexibility of the α7 helix, the root-mean-square deviation of atomic positions (RMSD) of heavy atoms was calculated along with the simulation. Compared to the extended-closed form, the α7 helix in the extended-open form shows higher RMSD, indicating that the α7 helix in the open form may possess higher flexibility (Figure 4A). Interestingly, leukadherin-1 shows little effect on the flexibility of the α7 helix in the extended-closed form, however, it decreases the flexibility of the α7 helix in the extended-open form, which is similar to the results from MSMs. Different from leukadherin-1, oleanolic acid increases the RMSD of the α7 helix along with the simulation indicating that it is able to increase its flexibility. Besides, oleanolic acid is able to increase the percentage of coils but to decrease the percentage of structured elements along with the simulation indicating the decreased secondary-structure stability in the α7 helix (Figure 4A). To further compare the effects of oleanolic acid and leukadherin-1 on the flexibility of the α7 helix, the secondary structure alteration along with the simulation was plotted. As shown in Figure 4B, leukadherin-1 shows little effect on the stability of the secondary structure in the α7 helix, whereas the well-organized alpha-helical structure was replaced with a 3-helix and bend along in the oleanolic acid/α_M_-I domain complex indicating that oleanolic acid may further increase the flexibility of the α7 helix.

### Oleanolic Acid Decreases the Free-Energy Requirement of the α7 Helix From the Extended-Closed Form to the Extended-Open Form and Stabilizes Its Extended-Open Conformation

**FIGURE 5:**
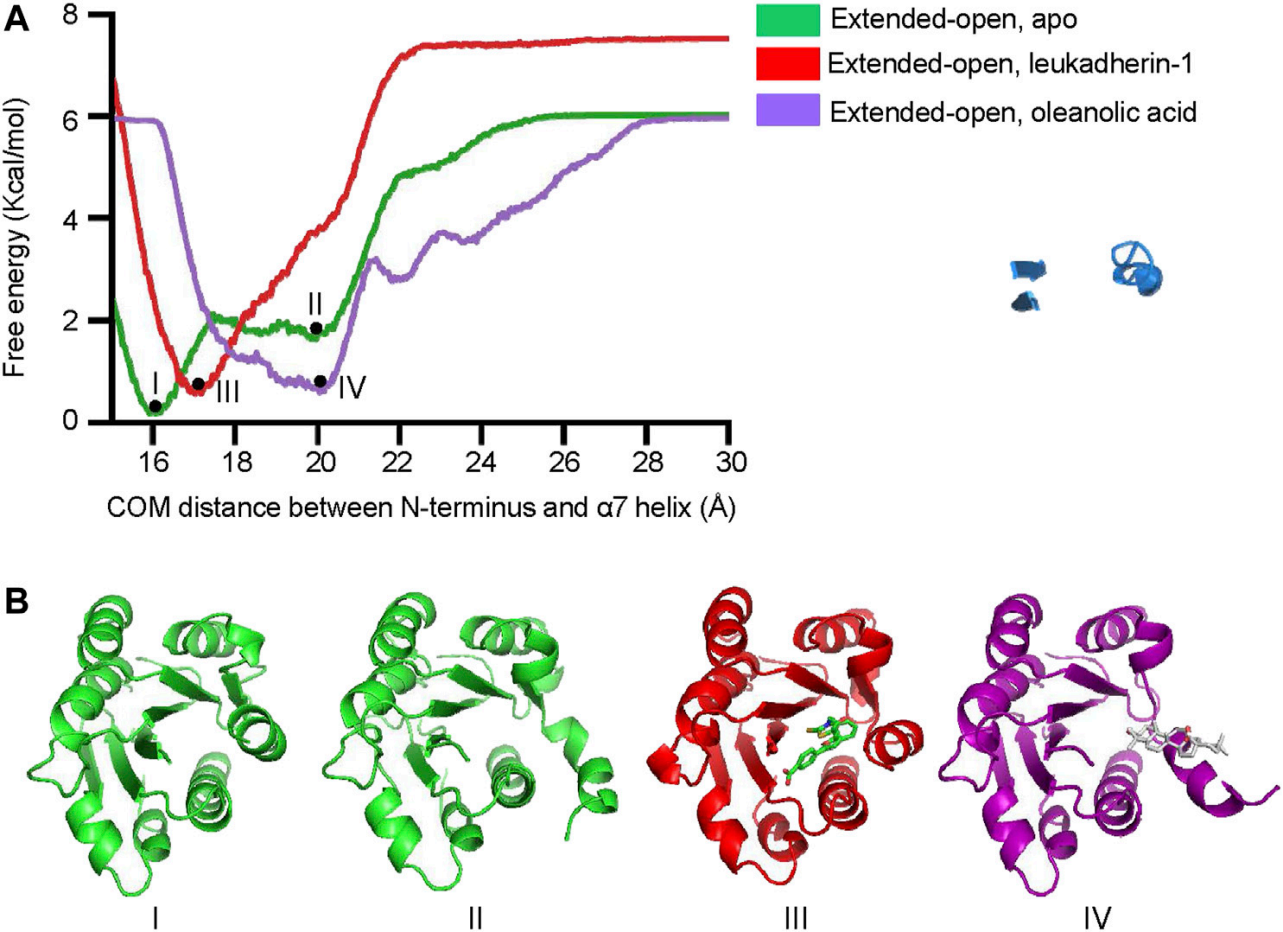
Oleanolic acid decreases the free-energy requirement of the α7 helix from extended-closed conformation to extended-open conformation. **(A)** The free energy profile of metadynamics simulation in five different models. The center of mass (COM) distance between N-terminus and the α7 helix was defined as collective variables (CV). Two local minima (I, II) were identified in apo form, while one local miminum was identified in complex with leukadherin-1 (III) and oleanolic acid (IV), respectively. **(B)** The representative structures of each local minimum are illustrated. The carbon atoms of leukadherin-1 and oleanolic acid are colored green and white, respectively.

To explore the free energy profile along with the conformational change of the α7 helix from extended-closed conformation to extended-open conformation, metadynamics simulation was employed, and the average free energy landscape for each simulation system was calculated from three independent metadynamic simulations (Supplementary Figure S6). In the apo-form, the α_M_-I domain showed two local minima (I and II), which represent the extended-closed and extended-open conformation, respectively (Figure 5A). The lower free energy difference between the two local minima indicated that the α_M_-I domain may reach an equilibrium between extended-open and extended-closed forms. In the presence of leukadherin-1, only one local minimum was detected (III), and the COM distance between the N-terminus and the α7 helix (16.9 Å) indicates that leukadherin-1 stabilizes the α_M_-I domain in its extended-closed conformation. Interestingly, only one local minimum was detected in the presence of oleanolic acid (IV), the relatively large distance between the N-terminus and the α7 helix indicates that the α_M_-I domain is stabilized in the extended-open conformation. Besides, we found that the free-energy requirement for inducing larger conformational shift of the α7 helix (COM distance larger than 22 Å) was lower in the presence of oleanolic acid indicating that oleanolic acid may facilitate the conformational change from the extended-closed to the extended-open conformation (Figure 5B **).**

### The Extended-Closed Conformation of the α7 Helix Is Partially Stabilized by the Α1 Helix

**FIGURE 6:**
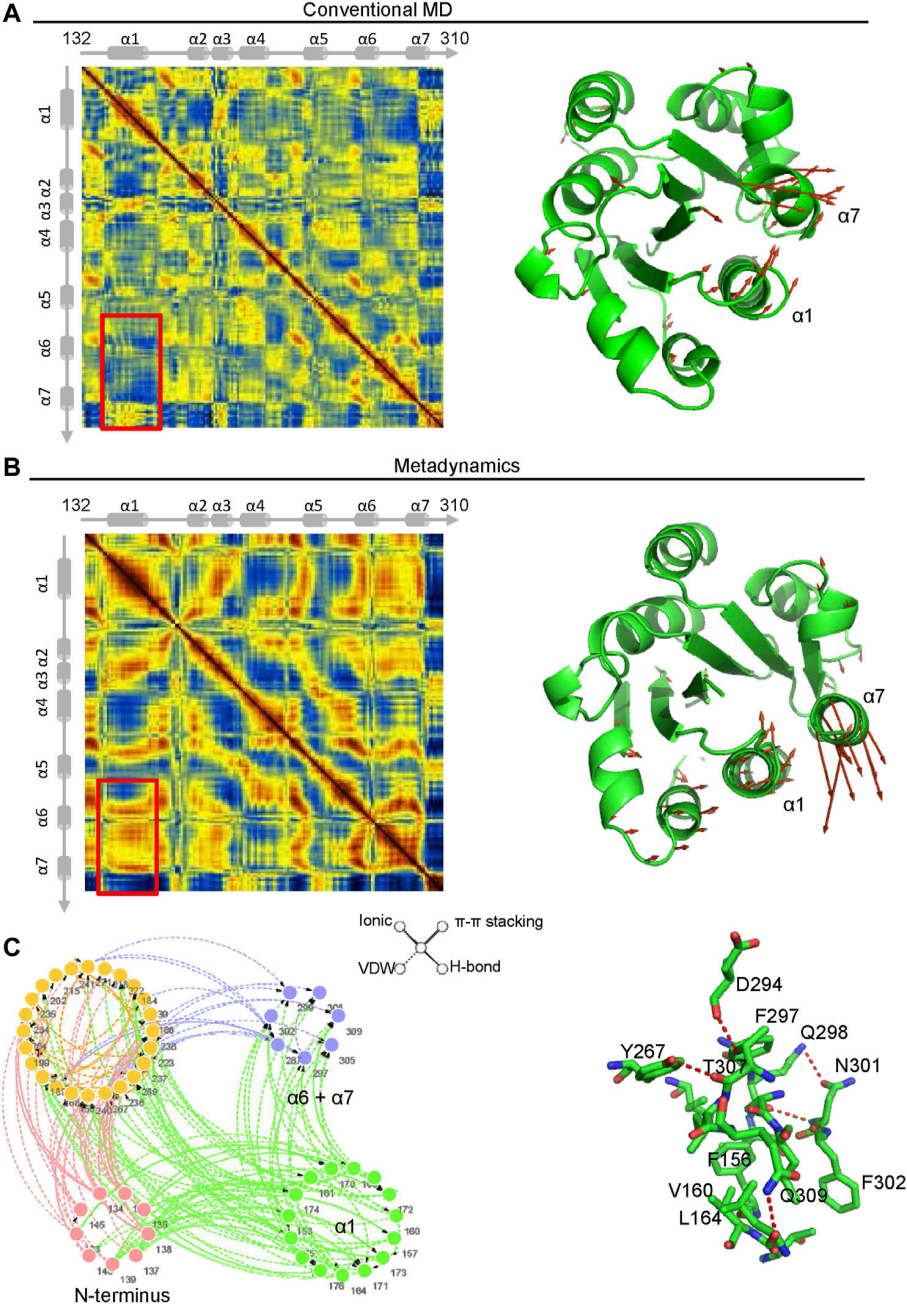
The interaction between the α1 and α7 helix contribute to stabilization of the α7 helix in its extended-closed form. Dynamics cross-correlation matrices (DCCMs) of the α_M_-I domain (left hand panel) in extended-open form under conventional molecular dynamics (MD) simulation (**A**) and metadynamics simulation **(B)**. The increased anti-correlated motion between the α1 and α7 helices was detected in metadynamics simulation. The directions of motion of Cα atoms are plotted with orange arrows (right hand panels). **(C)** Residue Interaction Network (RIN) analysis of residues in the α_M_-I domain. The occurrence of interactions greater than 5% is plotted in the left hand panel. The hydrogen bonds, vdW interactions, π-π stackings, and salt bridges are illustrated by solid lines, dotted lines, double lines, and contiguous arrows, respectively. A snapshot of interactions between residues in the α1 and α7 helices is shown in the right hand panel.

Dynamics Cross Correlation Maps (DCCMs) were employed to investigate the long-range interactions of the α_M_-I domain in extended-open conformations. Comparing the results from metadynamics simulation and conventional molecular dynamics simulation, a remarkably decreased correlated movement between α1 and α7 is observed, and the conversion from correlated movement to anti-correlated movement occurs as illustrated in the right hand panels (Figures 6A,B). To further investigate the molecular mechanism of how α7 is stabilized in its extended-closed form, residue interaction network analysis was employed, and the occurrence of interactions greater than 5% was extracted for further analysis. As shown in Figure 6C, a residue interaction network between the N-terminus, the α1 helix, and the α7 helix was detected, and the organized conformation of the α7 helix may partially stabilize through hydrogen bonds of the network between D294, T307, Y267, Q298, N301, and K165. Besides, the hydrophobic interactions between F156, V160, V164, and F302 may also contribute to stabilization of the α7 helix in its closed conformation.

### Oleanolic Acid Facilitates the Transformation of the α7 Helix From the Extended-Closed Form to the Extended-Open Conformation Partially Through Binding to the Pocket Between the α1 and α7 Helices

**FIGURE 7:**
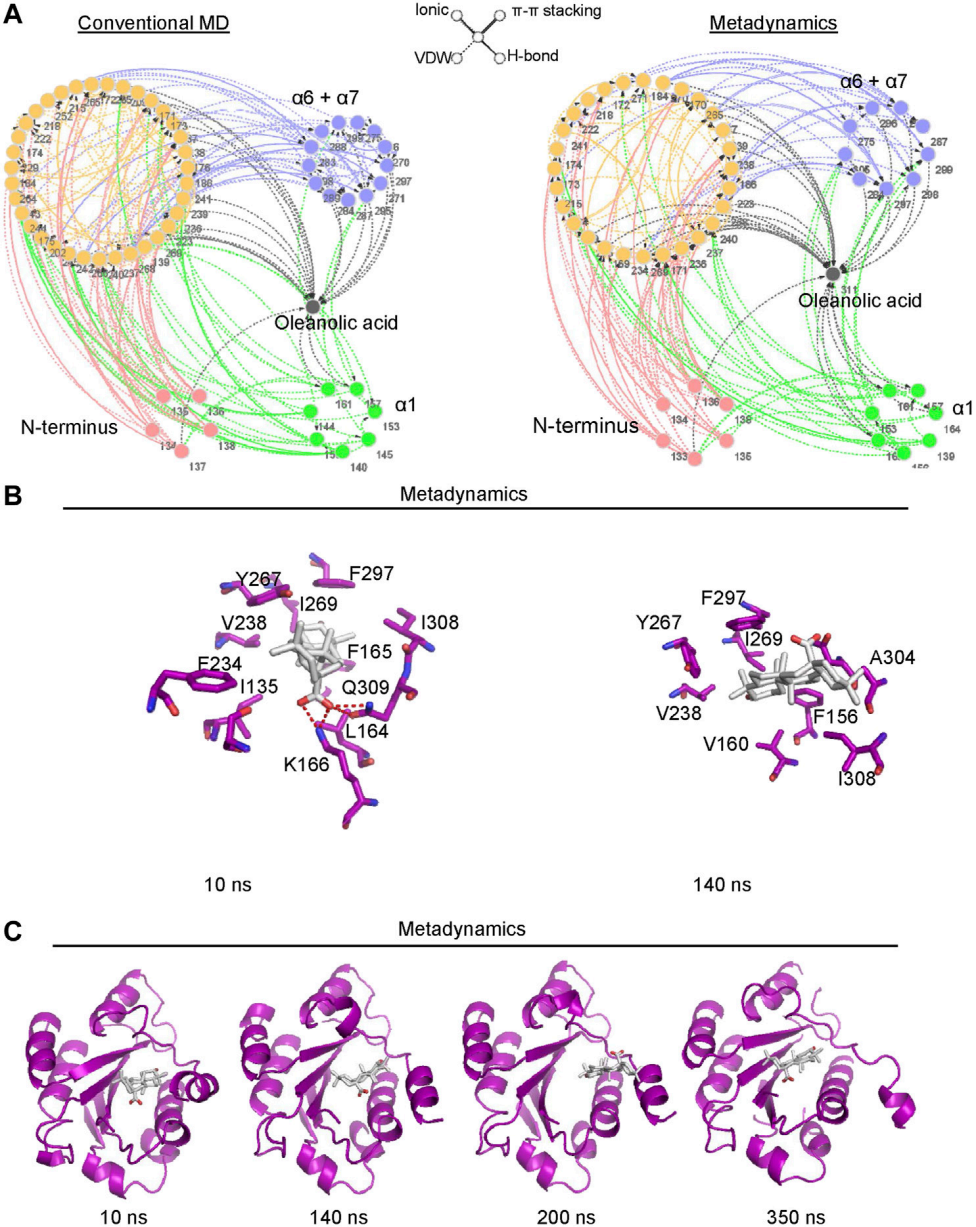
Metadynamics simulation identified a potential pocket for oleanolic acid formed by the α1 helix and the partially shifted α7 helix. **(A)** Residue Interaction Network (RIN) analysis identified an increased occurrence of interactions between oleanolic acid and residues in the α1 helix under metadynamics simulation. The occurrence of interactions greater than 5% is plotted. The hydrogen bonds, vdW interactions, π-π stackings, and salt bridges are illustrated by solid lines, dotted lines, double lines, and contiguous arrows, respectively. **(B)** Oleanolic acid falls into the binding pocket between the α1 and partially shifted α7 helices (from 10 to 200 ns), whereasα oleanolic acid escapes from the pocket, when the α7 helix is partially unfolded (350 ns). **(C)** The interactions between oleanolic acid and surrounding residues are shown. The carbon atoms of oleanolic acid are colored white.

To better understand the mechanism of conformational change of the α7 helix induced by oleanolic acid, the residue network of the α_M_-I domain/oleanolic acid complex was analyzed using conventional molecular dynamics simulation and metadynamics simulation. Compared with the residue interaction network of the α_M_-I domain in its apo form, oleanolic acid significantly decreased the direct interaction between the N-terminus or the α1 helix and the α7 helix (Figure 7A). Although, oleanolic acid may serve as a connector to bridge the α7 helix and other parts of the α_M_-I domain, the steric repulsion between steroid scaffold and the residues with bulky side chain, such as V160, R152, M154, I269, F297, and V238, may force the α7 helix to move away from its original conformation (Figure 7B). This finding is also supported by the trajectory of metadynamics simulation (Figure 7C). By analyzing the trajectory of metadynamics simulation, we found that oleanolic acid not only shifted the α7 helix away from the N-terminus, but also induced the unfolding of the α7 helix during the simulation (350 ns snapshots in Figure 7C).

## Discussion

Oleanolic acid is an important bioactive ingredient in a variety of medicinal plants with incompletely understood molecular targets. The data of our study provide evidence that oleanolic acid may function as an allosteric agonist of integrin α_M_ through binding to its α_M_-I domain.

Importantly, the membrane distribution of integrins is closely controlled by their conformation, and it has been proposed that formation of integrin clusters is a result of conformational transformation from the closed to the extended-open conformation (Welf et al., 2012). Although, the clustering of inactive integrins have also been detected, they are less organized and more dispersed (Michael and Parsons, 2020). In our study, we found that oleanolic acid is able to induce the clustering of integrin α_M_ on human macrophages, which is comparable to that induced by the α_M_ activator fibrinogen. This suggests that oleanolic acid may induce conformational change of integrin α_M_ from the closed (bent-closed or extended-closed) dominant to the extended-open dominant conformation.

In response to biomolecules, such as intercellular adhesion molecule family members, complement protein iC3b and fibrinogen, the metal ion-dependent association site (MIDAS) within the α_M_-I domain of integrin α_M_ recognizes the ligand and binds it to the aspartate in its motif with high affinity (Mould et al., 2002; Bajic et al., 2013; Liddington, 2014). The binding of a carboxyl group of an acidic residue or a ligand to the MIDAS within the α_M_-I domain is able to cause a switch in Mg^2+^ coordination, and finally leads to a 10 Å downward movement of the α7 helix located in the C-terminus. The shift of the α7 helix was considered to be highly significant, as it may further cause the re-arrangement of the propeller and β-I domain, and initiate the conformational change and activation of integrin α_M_ (Mould et al., 2002; Liddington, 2014). Finally, the randomly distributed α_M_ on the cell membrane will form clusters and initiate signal transduction in the down-stream signaling pathway (Lim and Hotchin, 2012).

As MIDAS plays an essential role in the activation of integrin α_M_, this motif was considered as the first candidate as oleanolic acid binding site. However, the crystal structure of the complex between α_M_-I domain and biomolecules demonstrated that besides the electrostatic selectivity, MIDAS imposes strict steric selectivity on its ligands. Thus, a carboxyl group connected to longer side chains, such as aspartate or glutamate, represents an ideal MIDAS ligand (Bajic et al., 2013; Jensen et al., 2016). However, the carboxyl group of oleanolic acid is directly linked to the steroid scaffold causing a strong steric repulsion between steroid scaffold and surrounding residues that might affect the coordination between oleanolic acid and Mg^2+^ ions. As a result, the allosteric pocket, which is located around the α7 helix and which is able to bind a variety of molecules with distinct shape and chemical properties (Bjorklund et al., 2006; Faridi et al., 2009) was selected as the putative binding site for oleanolic acid. Our results from molecular docking and molecular dynamics simulation confirmed that oleanolic acid can be accommodated into the allosteric pocket at a similar binding site and with a similar predicted binding affinity as leukadherin-1, an integrin α_M_ ligand.

Interestingly, the results from molecular dynamics simulation showed that the effects of oleanolic acid and leukadherin-1 on α_M_-I domain differ remarkably. As demonstrated by *in vitro* studies, leukadherin-1 functions as an allosteric agonist of the α_M_-I domain by stabilizing it in extended-closed form (Faridi et al., 2013). In accordance with literature data, the results of our simulation showed that leukadherin-1 did not affect the overall stability of the α_M_-I domain nor the orientation of the α7 helix. By contrast, oleanolic acid was able not only to increase the distance between the N-terminus and the α7 helix, but also to induce the partial unfolding of the α7 helix, which is similar to the conformational switch caused by an I316G mutation (Xiong et al., 2000). The above mentioned observation indicates that oleanolic acid is able to destabilize the α7 helix of the α_M_-I domain in its closed form.

Additionally, our results show that oleanolic acid can facilitate the conformational switch of integrin α_M_ from the closed to the open form by altering the free-energy profile. It is widely accepted that integrins exist in a continuous conformational equilibrium ranging from a compact bent-closed form (organized α7 helix, shorter distance between the N- and the C-termini) to a fully extended open form (less stable α7 helix and shifted C-terminus) (Askari et al., 2009; Hanein and Volkmann, 2018). In accordance with literature data, our results from metadynamics simulation showed that the α_M_-I domain in its apo form possesses two local minima. A more stable state is characterized by the smaller distance between the N- and C-termini resembling the bent-closed state. A less stable state is characterized by the larger distance between N- and C-termini and a partially unfolded α7 helix resembling the extended-open state. However, only one local minimum was observed in the presence of oleanolic acid, which is characterized by a larger distance between N- and C-termini resembling the extended-open state. The data from residue interaction network analysis further demonstrated that the interrupted interaction between the α7 helix and α1 or the N-terminus may contribute to the conformational shift of the α7 helix in the presence of oleanolic acid. The combinatorial effects of destabilizing the α7 helix in its closed form and stabilizing the α7 helix in its open form may finally lead to the shifted equilibrium to the extended-open form, and provide a plausible explanation for the observed integrin α_M_ clustering after oleanolic acid treatment. Interestingly, α_M_-I domain/leukadherin-1 complexes also showed a remarkably different free-energy profile than the α_M_-I domain in apo form and α_M_-I domain/oleanolic acid complex. Only one local minimum with a slightly increased distance between the α7 helix and the N-terminus was observed indicating that leukadherin-1 may stabilize the α7 helix in an extended-closed state, which was commonly considered as an intermediate between bend-closed and extended-open state (Hogg et al., 2011).

Though β_2_ integrin also contains an I-like domain that is structurally similar to the α_M_-I domain with a confirmation that can be changed upon ligand binding, integrin ligands bind to the α-I domain and it is the α subunit of an integrin heterodimer that regulates binding affinity and specificity of different ligands (Springer and Dustin, 2012). Yet, ligand binding to the β_2_-I-like domain act as α_M_β_2_ antagonists blocking the α_M_-I domain in a low energy, inactive, and closed conformation (Shimaoka and Springer, 2003). In contrast, oleanolic acid has the opposite effect and induced integrin α_M_β_2_ clustering supporting our hypothesis that it targets the α_M_-I domain.

In summary, the results from our study show that the allosteric pocket of α_M_-I domain within integrin α_M_ presents a potential target of oleanolic acid. Combining *in silico* simulation and *in vitro* experiments, we found that oleanolic acid is able to induce clustering of integrin α_M_ partially through destabilizing the α7 helix in its closed form and stabilizing the α7 helix in its partially unfolded state. The conformational change is facilitated by lowering the free-energy barrier between the two states, and finally shifting the balance from closed to open form. Although we have found that oleanolic acid induces the clustering of integrin α_M_ and decrease macrophage motility similar to the α_M_ agonists leukadherins, the conformational alteration of α_M_ caused by oleanolic acid may not be sufficient to induce full activation of integrin α_M_β_2_ as described for its physiological ligand fibrinogen.

## Data Availability

The original contributions presented in the study are included in the article/Supplementary Material, further inquiries can be directed to the corresponding author.
