# Supplementary material for: Identification of Oleanolic Acid as Allosteric Agonist of Integrin αM by Combination of In Silico Modeling and In Vitro Analysis
Source: Front Pharmacol. 2021 Sep 17;12:702529. doi: 10.3389/fphar.2021.702529 (PMC8484648; doi:10.3389/fphar.2021.702529)
Supplement: Supplementary file 1 [file DataSheet1.PDF]

Supplementary Material

1 Supplementary Methods

1.1 Predicted binding free energy between I domain of α_M integrin (α_M-I) and ligands by molecular mechanics/Poisson-Boltzmann surface area (MM-PBSA) analysis

The MM-PBSA protocol (Zhu *et al.*, 2020) was used to calculate the free energy of binding using the MMPBSA.py.MPI module (Miller *et al.*, 2012). Based on the last 100 ns of MD trajectory, 2000 snapshots every 50 ps were selected and the binding free energy within the complexes was calculated. Then, a decomposition of binding free energy was performed and the contributions from the residues located at the interaction interface were calculated.

2 Supplementary Figures and Tables

2.1 Supplementary Figures

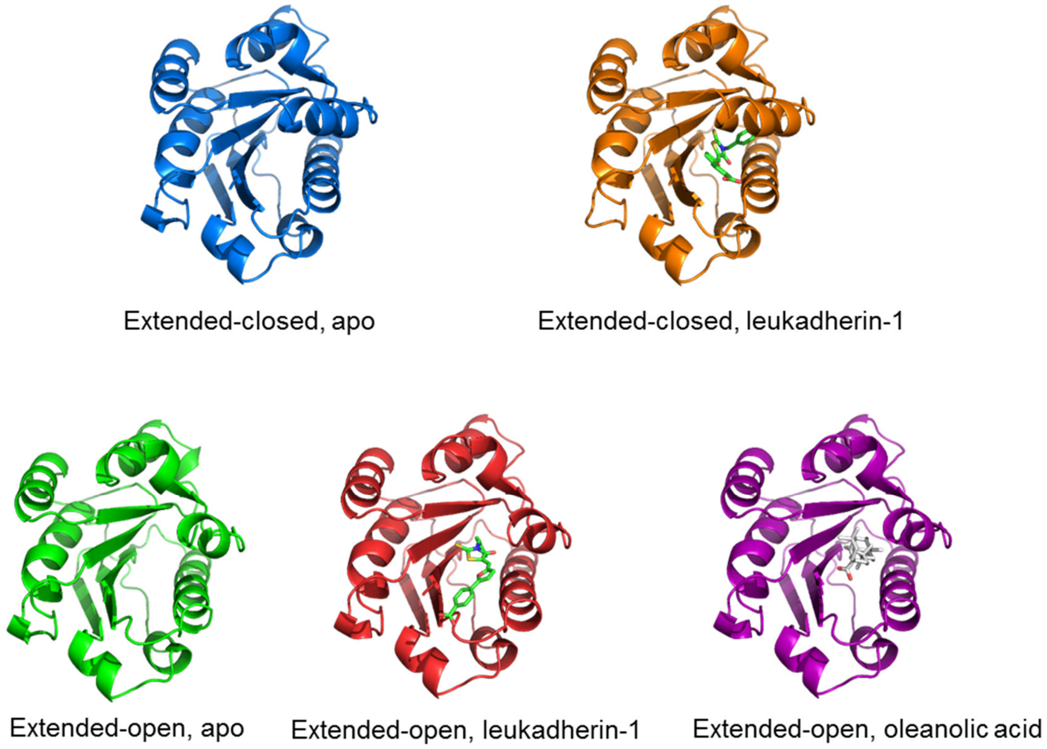

Supplementary Figure S1 | Starting α_{M}-I domain structures for molecular dynamics simulations. The carbon atoms of leukadherin-1 and oleanolic acid are colored green and white, respectively.

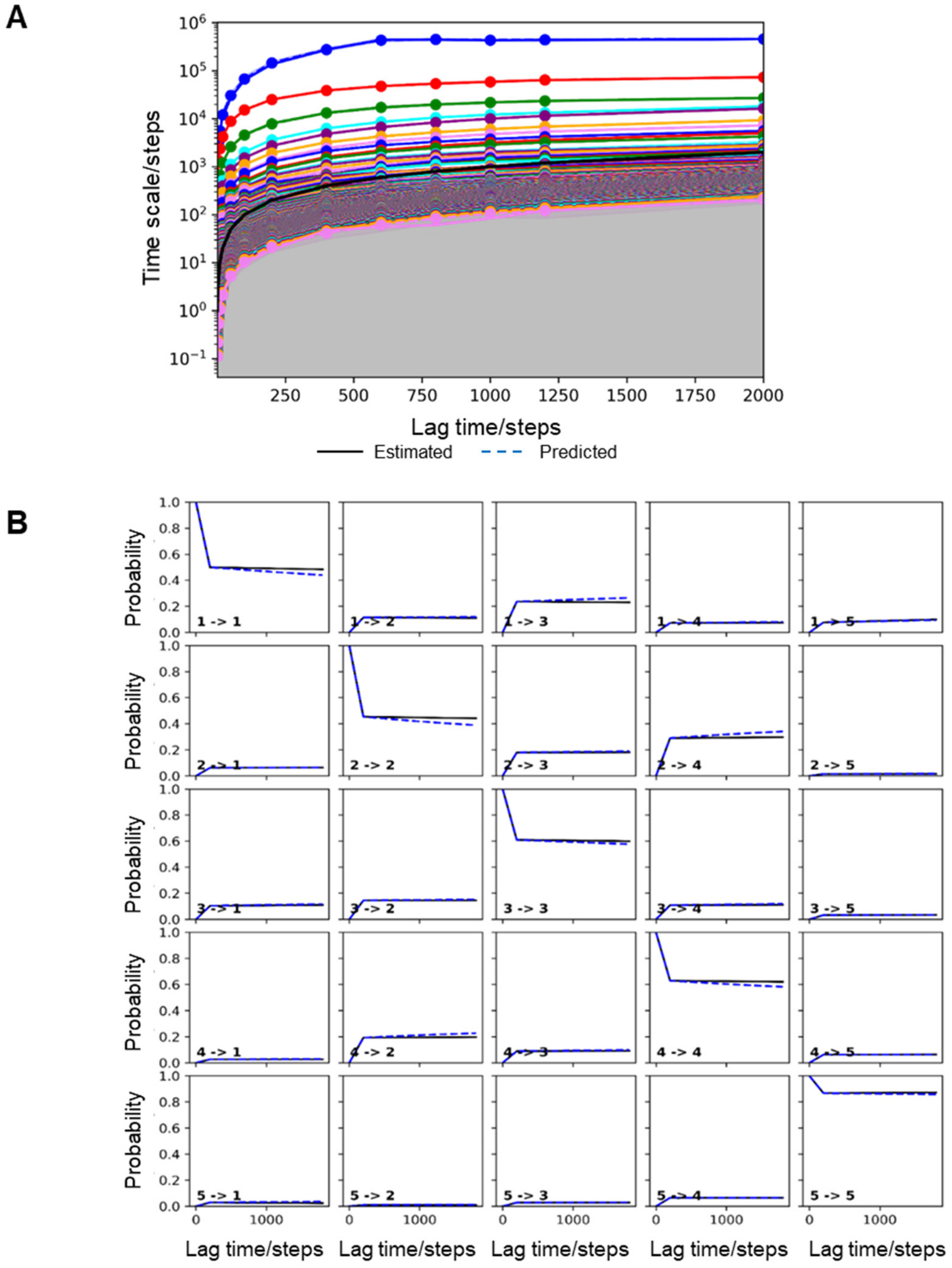

Supplementary Figure S2 | Lag time estimation and Chapman-Kolmogorov test for Markov state modeling of the α_M-I domain. **(A)** Lag time estimation shows a constant time scale after t = 5.0 ns (1000 steps). **(B)** Chapman-Kolmogorov test demonstrates that the lag time = 5.0 ns (1000 steps) and the chosen states number (5 states) are suitable for Markov state model (MSM) construction. Lag time estimates are shown as black lines, predicted estimated errors are shown as gray shadows**.**

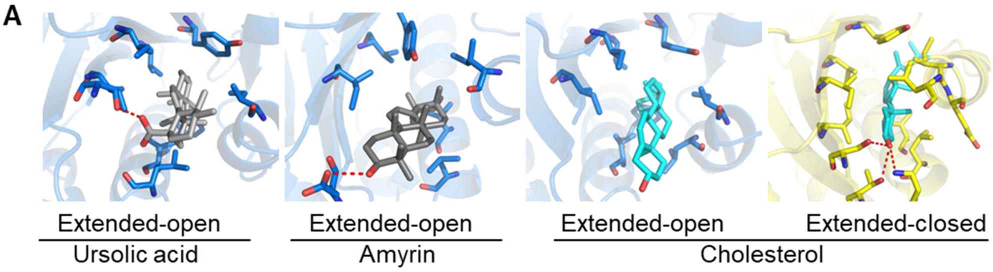

B

	Oleanolic acid	Ursolic acid	Amyrin	Cholesterol
**Extended-closed** (PDBID: 1IDO)	N.D	N.D	N.D	-13.67
**Extended-open** (PDBID:4M76)	-9.03	-7.25	-8.96	-8.82

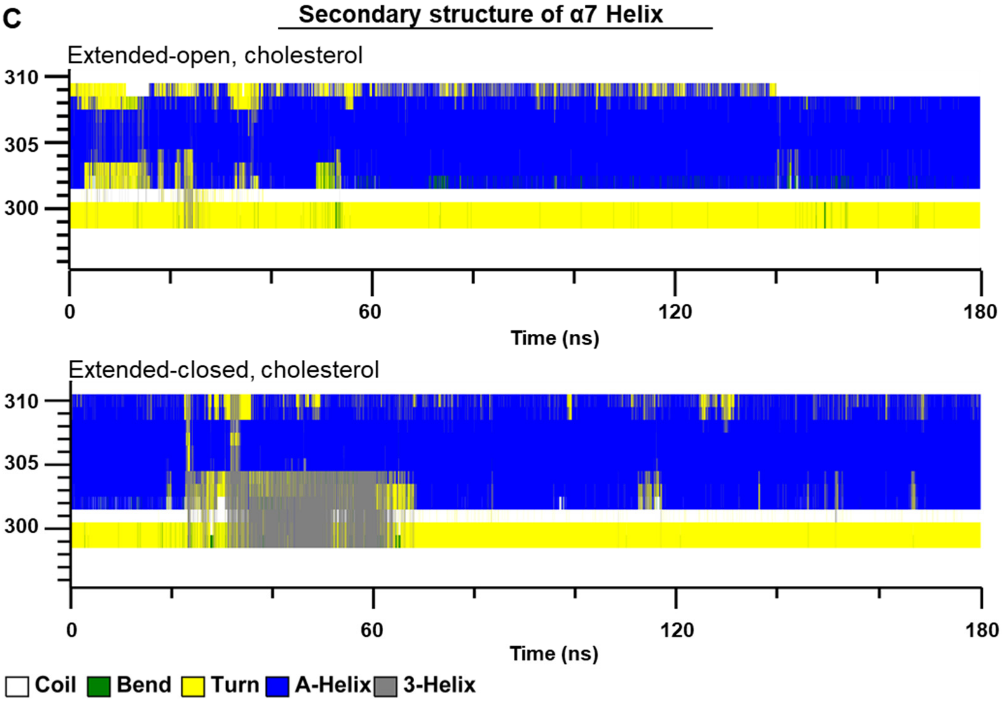

Supplementary Figure S3 | Comparison of binding of oleanolic acid, ursolic acid, amyrin, and cholesterol to the α_M-I domain as analyzed by molecular docking and molecular dynamics simulation. (**A**) The top-scored binding poses of ursolic acid (grey sticks), amyrin (grey sticks), and cholesterol (cyan sticks) at the allosteric site of the α_M-I domain in its extended-open and extended-closed conformations. Helices and sheets of the α_M-I domain are light blue (extended-open conformation) or light yellow (extended-closed conformation); amino acid residues in the proximity to the compounds are blue or yellow for extended-open and extended-closed conformation, respectively. (**B**) The predicted binding free energy of oleanolic acid, ursolic acid, amyrin, and cholesterol to the α_M-I domain. (**C**) Cholesterol does not affect the stability of the secondary structure of the α7 helix as analyzed by conventional molecular dynamics simulation.

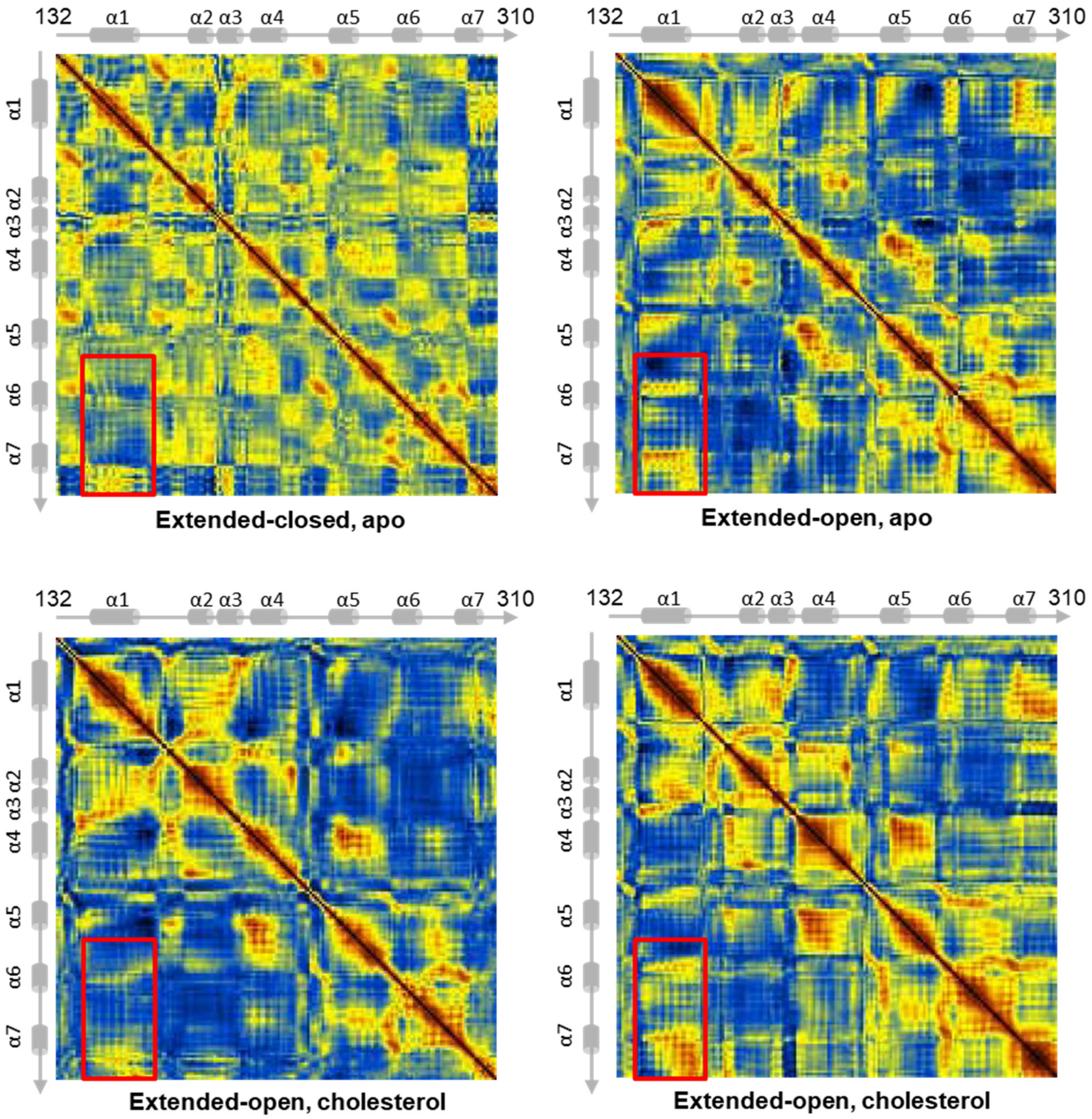

Supplementary Figure S4 | Cholesterol exhibits negligible effects on the direction of the α7 helix movement. Dynamics cross-correlation matrices (DCCMs) of the α_M-I domain in extended-closed and extended-open conformations in the absence (upper panels) and in the presence (lower panels) of cholesterol as analyzed by conventional molecular dynamics (MD) simulation. Red rectangles mark the areas with small changes in motion between the α1 and α7 helices in the presence of cholesterol.

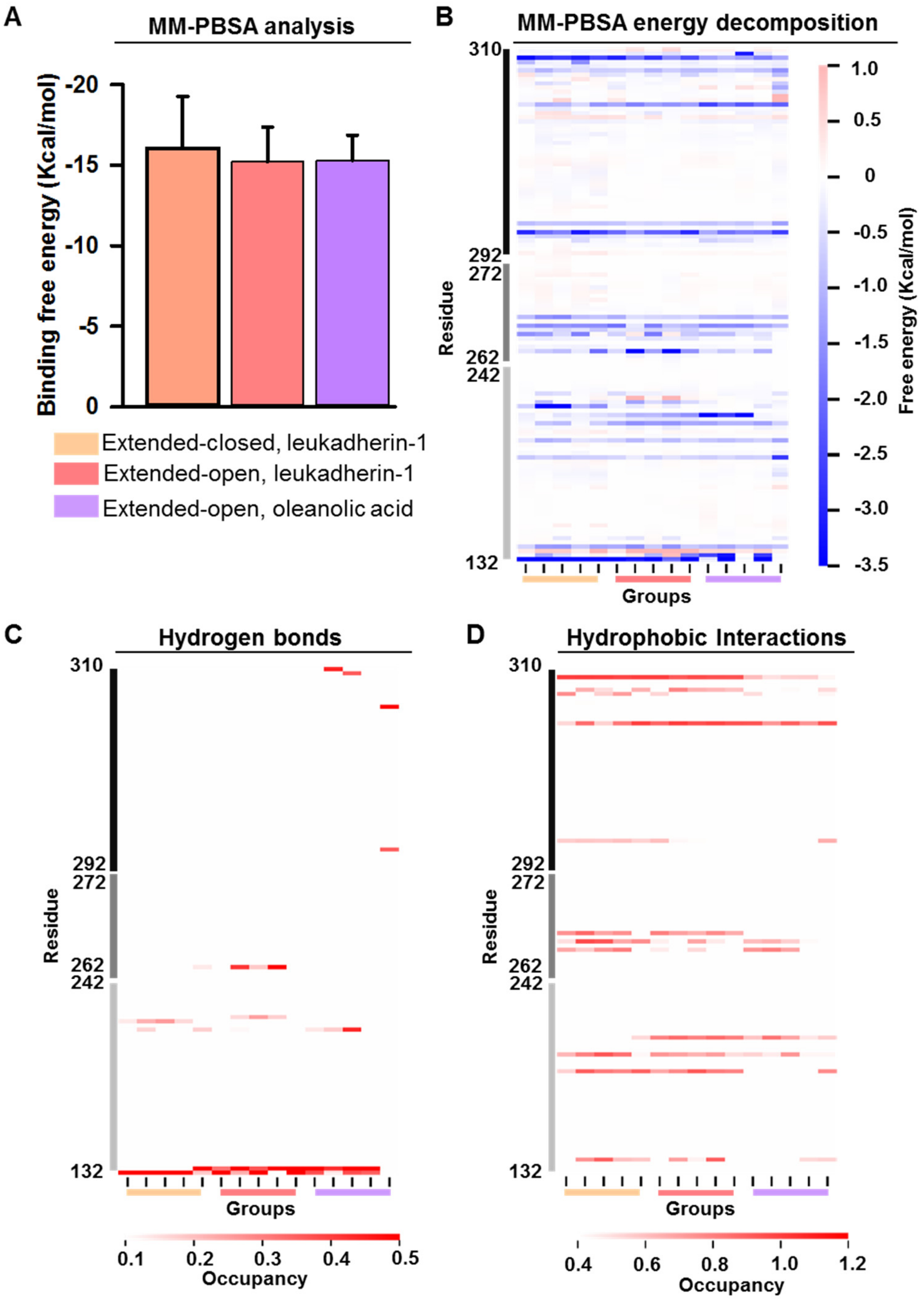

Supplementary Figure S5 | Leukadherin-1 and oleanolic acid exhibit comparable potential binding affinities to the α_M-I domain that are mainly stabilized by hydrophobic interactions. (A) The binding free energy as predicted by MM-PBSA. The data are presented as mean ± SEM of five independent simulations. (B) Free energy decomposition of residues in each independent simulation. The occupancy of hydrogen bonds and hydrophobic interactions are plotted in (C) and (D), respectively. The occurrence of hydrogen bonds or hydrophobic interactions in each independent experiment are plotted in separate columns. The color bars below the columns illustrate the respective group: orange for the α_M-I domain extended-closed, leukadherin-1; red for the α_M-I domain extended-open, leukadherin-1; purple for the α_M-I domain extended-open, oleanolic acid.

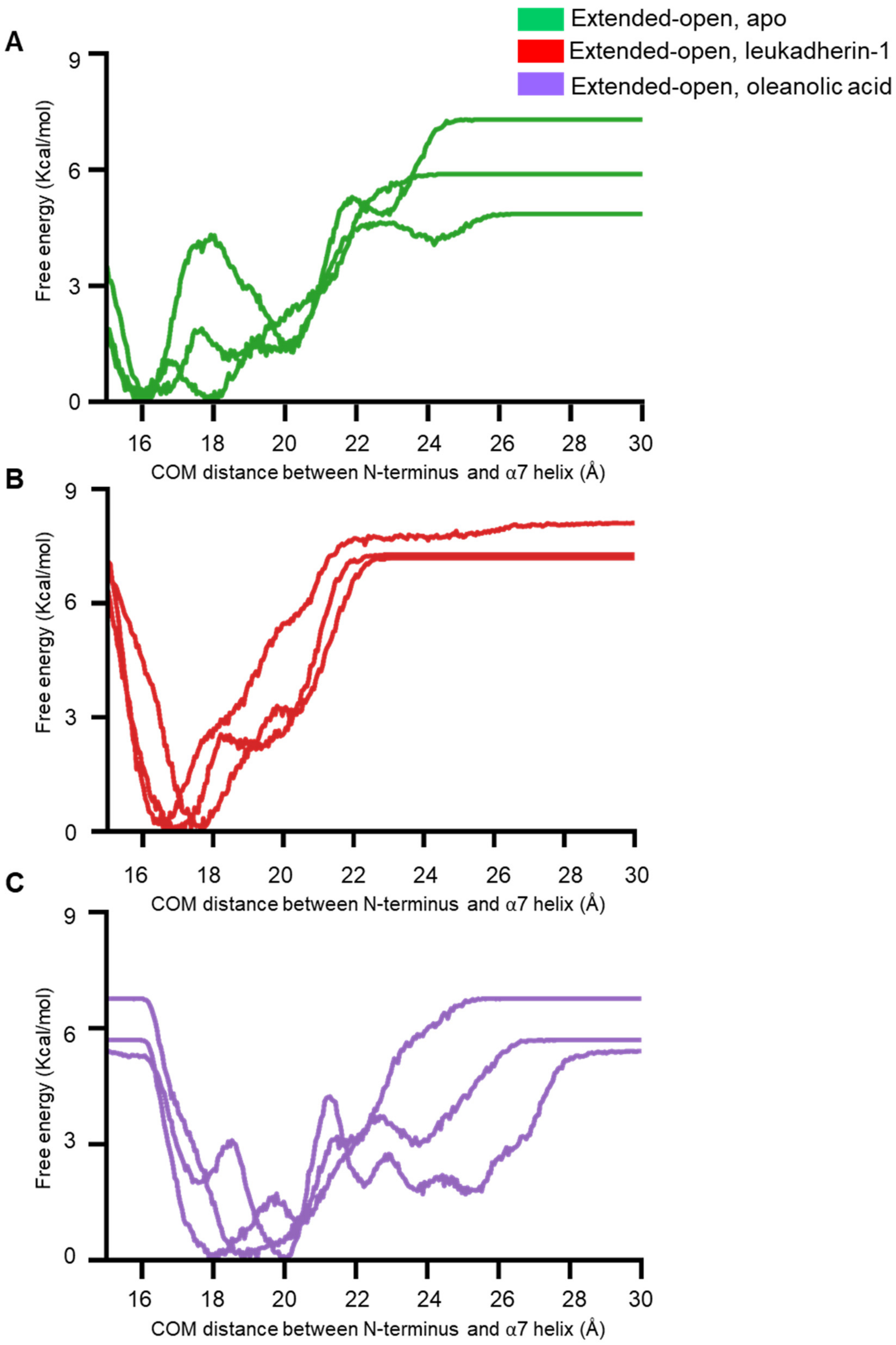

Supplementary Figure S6│ Results from three independent metadynamics simulations of the α_M-I domain. **(A)** Extended-open form, apo. **(B)** Extended-open form with leukadherin-1. **(C)** Extended-open form with oleanolic acid.

2.2 Supplementary Table

TABLE S1 | Summary of all simulations.

Simulation of α_M-I domain	Type	Replicates	Total simulation time (ns)
**Extended-closed, apo**	Conventional MD	5	2632.456
**Extended-closed, leukadherin-1**	Conventional MD	5	2548.276
**Extended-open, apo**	Conventional MD	5	2670.782
**Extended-open, leukadherin-1**	Conventional MD	5	2668.557
**Extended-open, oleanolic acid**	Conventional MD	5	2738.662
**Extended-open, apo**	Metadynamics	3	1937.855
**Extended-open, leukadherin-1**	Metadynamics	3	1910.364
**Extended-open, oleanolic acid**	Metadynamics	3	1797.273
**Extended-open, Cholesterol**	Conventional MD	3	544.655
**Extended-closed, Cholesterol**	Conventional MD	3	551.734

3 References

Miller B.R., 3rd, McGee T.D., Jr., Swails J.M., Homeyer N., Gohlke H., and Roitberg A.E. (2012). MMPBSA.py: An efficient program for end-state free energy calculations. *J. Chem. Theory Comput.* 8, 3314-21. doi:10.1021/ct300418h.

Zhu K., Du D., Yang R., Tao H., and Zhang H. (2020). Identification and assessments of novel and potent small-molecule inhibitors of EED-EZH2 interaction of polycomb repressive complex 2 by computational methods and biological evaluations. *Chem. Pharm. Bull. (Tokyo)* 68, 58-63. doi:10.1248/cpb.c19-00550.
